# Proteomic Analysis of Non-depleted Serum Proteins from Bottlenose Dolphins Uncovers a High Vanin-1 Phenotype

**DOI:** 10.1038/srep33879

**Published:** 2016-09-26

**Authors:** Philip Sobolesky, Celeste Parry, Baylye Boxall, Randall Wells, Stephanie Venn-Watson, Michael G. Janech

**Affiliations:** 1 Division of Nephrology, Department of Medicine, Medical University of South Carolina, Charleston, SC, 29425, USA; 2 Translational Medicine and Research Program, National Marine Mammal Foundation, San Diego, CA 92106, USA; 3 Grice Marine Laboratory, Department of Biology, College of Charleston, Charleston, SC 29412, USA; 4 Chicago Zoological Society’s Sarasota Dolphin Research Program, c/o Mote Marine Laboratory, Sarasota, FL 34326, USA

## Abstract

Targeted approaches have been widely used to help explain physiological adaptations, but few studies have used non-targeted omics approaches to explore differences between diving marine mammals and terrestrial mammals. A rank comparison of undepleted serum proteins from common bottlenose dolphins (*Tursiops truncatus*) and pooled normal human serum led to the discovery of 11 proteins that appeared exclusive to dolphin serum. Compared to the comprehensive human plasma proteome, 5 of 11 serum proteins had a differential rank greater than 200. One of these proteins, Vanin-1, was quantified using parallel reaction monitoring in dolphins under human care and free-ranging dolphins. Dolphin serum Vanin-1 ranged between 31–106 μg/ml, which is 20–1000 times higher than concentrations reported for healthy humans. Serum Vanin-1 was also higher in dolphins under human care compared to free-ranging dolphins (64 ± 16 vs. 47 ± 12 μg/ml P < 0.05). Vanin-1 levels positively correlated with liver enzymes AST and ALT, and negatively correlated with white blood cell counts and fibrinogen in free-ranging dolphins. Major differences exist in the circulating blood proteome of the bottlenose dolphin compared to terrestrial mammals and exploration of these differences in bottlenose dolphins and other marine mammals may identify veiled protective strategies to counter physiological stress.

In mammals, evolutionary adaptations have permitted different strategies for survival thereby allowing species to exploit a diverse array of environments. Many of these strategies are considered detrimental within the confines of normal human physiology and are consistent with the promotion of significant organ injury^1^. Marine mammals are similar in this regard in that some have evolved physiological and morphological adaptations that permit rigorous dives, repeated bouts of hypoxia, little access to free-water, and long periods of fasting^2^,^3^,^4^,^5^. Comparative studies demonstrated that diving leads to greater reactive oxygen species burden^6^, likely due to repeated peripheral vasoconstriction and organ reperfusion^7^,^8^,^9^,^10^. In response, there is a far greater need to oppose oxidative stress and this response is reflected in the elevation of antioxidant enzyme activity in tissues^11^,^12^,^13^ (*e.g.* glutathione disulfide reductase, superoxide dismutase, glutathione peroxidase), and red blood cells^6^ (*e.g.* glutathione reductase, glutathione peroxidase, superoxide dismutase and catalase). Biochemical adaptations to hypoxia include high muscle myoglobin content^14^, expression of cytoprotective neural globins^3^, an elevation in numbers of circulating red blood cells, and high per unit mass hemoglobin concentration^14^. Furthermore, bottlenose dolphins and killer whales (*Orcinus orca*) have low levels of circulating clotting factors VII and X, and a complete lack of Hageman factor (XII) which may help to prevent thrombus formation during episodes of peripheral vasoconstriction^15^.

In marine mammals, the high glucose demands of red blood cells and tissues can require elevated glucose production or cycling during long periods of fasting in some species^16^. Fasted bottlenose dolphins exhibit higher serum glucose concentrations compared to horses and cows^17^ and slightly higher plasma glucose levels than fed dolphins^18^ suggestive of a natural “diabetic-like” state that may be beneficial during times of short-term fasting. Long-term fasting hyperglycemia and insulin resistance is a hallmark of fasting Northern elephant seals^19^ and evidence of insulin-independent glucose uptake^20^,^21^ suggests that hormonal regulation of glucose metabolism has deviated significantly from terrestrial mammals.

Many insightful studies of marine mammal physiology and health have focused on oxygen transport, antioxidant, metabolic, and clinical laboratory-relevant proteins in the blood; however, comparative studies describing the circulating serum proteome in marine mammals have not been extensively interrogated for novel serum phenotypes that could underlie significant alternative mechanisms of protection and/or metabolic regulation. To extend the discovery of phenotypic determinants in the serum that may promote the ability of marine mammals to counter stress, we created a normalized spectral abundance factor rank-list of high abundance serum proteins from bottlenose dolphins (*Tursiops truncatus*)^22^ alongside undepleted human and pig serum proteomes generated in-house. Outlier ranked proteins were listed and one of the outlier proteins, Vanin-1, was chosen to validate the rank differences. Measurements of Vanin-1 levels in both dolphins under human-care and free-ranging dolphins were made and correlated with clinical blood laboratory values.

## Results

### Serum rank comparisons: dolphin vs. human and pig

Analysis of tryptic protein digests identified 58 proteins in dolphin serum, 92 proteins in human serum, and 77 proteins in pig serum (Supplemental Table 1). Albumin was the highest ranked protein in each group. We utilized terms to describe the proteins as high, medium, or low based on previous descriptions of blood proteins^23^,^24^, where high abundance protein relates to a protein concentration between 50,000–1 mg/L; medium abundance protein relates to a protein concentration between 1–0.5 mg/L, and low abundance protein relates to a protein concentration less than 0.5 mg/L. Proteins were classified based on estimated human plasma protein concentration^25^. The use of the term, high abundance, to describe serum proteins in dolphins is based on the fact that these proteins were identified from tryptic peptides from sera that were not depleted of major proteins. Further, tryptic peptides were not fractionated by orthogonal methods and proteins were identified from a single LC/MS/MS run with data dependent acquisition.

**Figure 1:**
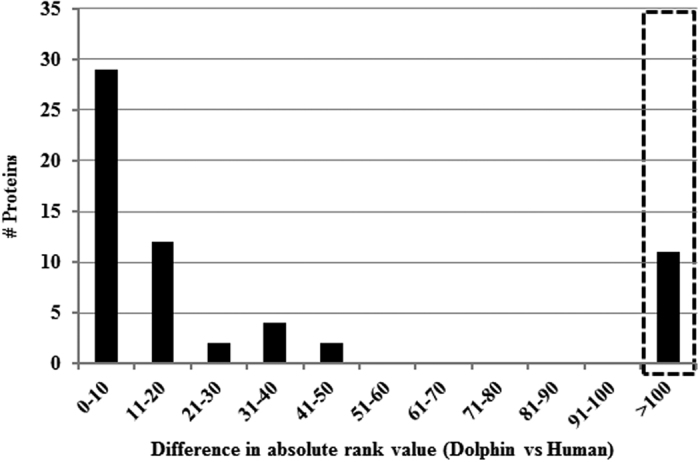
Histogram plot of difference between in mean protein rank between dolphin serum proteins and pooled normal human serum proteins. Absolute value of differences are plotted in bins of 10. Dashed box indicates 11 serum proteins that were not found in the pooled human serum proteomic dataset. Additional comparative ranking data and identifications are listed in Table 1.

There were 11 dolphin serum proteins that did not match any protein on the list of 92 high abundance human serum proteins (Fig. 1). The 11 unmatched ranked dolphin protein identifications are provided in Table 1. For dolphin proteins that did not match human serum proteins, a second comparison was made with pig serum proteins to determine whether phylogenetic differences in serum proteins could simply explain the differences between dolphin and human. Three out of 11 proteins that were considered unique to the dolphin high abundance serum proteome were ranked within the high abundance pig serum proteome. Fetuin B, Extracellular matrix protein 1, and Glycosylphosphatidylinositol phospholipase D1 were all detected in undepleted pig serum. The initial 11 outlier dolphin proteins were also compared to the high confidence human plasma reference proteome^25^ to provide further reference with estimated abundance in a well-characterized mammalian proteome. Although Fibrinogen beta chain and gamma chain proteins were not discovered in the initial survey of human serum proteins, the dolphin Fibrinogen chains (rank: 44 ± 19 and 47 ± 17, respectively) were ranked near the top of the human reference plasma proteome (rank 18 and 21). Similar to fibrinogen chains, dolphin glutathione peroxidase 3 appears to be an outlier compared to the initial human and pig serum protein survey produced from this study; however, compared to the reference human plasma proteome, dolphin Glutathione peroxidase 3 protein is only moderately different in terms of rank (27 ± 4 in dolphin vs. 69 in human). The remaining five out of 11 dolphin serum proteins, which were not observed in the initial human and pig serum proteome analysis and had a differential rank greater than 200 compared to the reference human proteome, were: Adiponectin, Vanin-1, Polymeric immunoglobulin receptor, Adipocyte plasma membrane protein, and Deleted in malignant brain tumors 1. Of these five proteins, only adipocyte plasma membrane protein was not identified in every dolphin in at least one time point (Table 1).

### Enrichment analysis

Of the 11 proteins in Table 1, seven proteins were common to high-level gene ontology biological process categories: Response to stress and Response to chemical stimuli (Supplemental Table 3). A third category, Regulation of immune system process, contained six of 11 proteins. All proteins were classified as cellular component: extracellular exosome. Evidence for cell-specific expression of human protein orthologs revealed 9 of 11 proteins are expressed in adipocytes (Supplemental Table 4). Vanin-1 and Extracellular matrix protein were not contained in the cell expression enrichment.

### Serum Vanin-1 measurements

**Figure 2:**
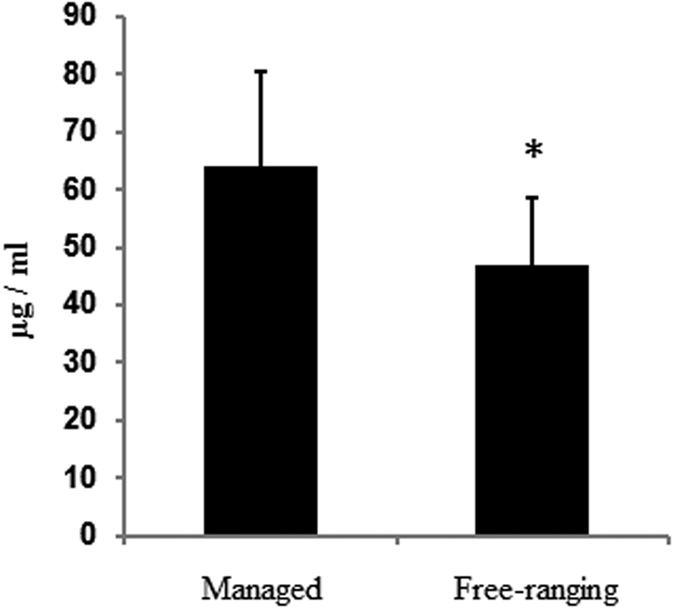
Vanin-1 concentration in the serum of managed or free-ranging bottlenose dolphins. *Indicates P < 0.05 versus managed dolphins. Bars represent mean vanin-1 serum concentration. Error bars indicate standard deviation.

A Vanin-1 assay was developed with a calculated limit of detection of 2.5 μg/ml and a calculated lower limit of quantification of 4.5 μg/ml. The highest intra-run variability was 2.5% RSD. Twenty dolphin samples (10 human-care and 10 free-ranging) were strictly age- and sex-matched from a previously published cohort^26^. Clinical laboratory data for 10 human-care dolphins (age range 4–29 years) and 10 free-ranging dolphins (age range 2–28 years) are listed in Table 2. Serum concentration of Vanin-1 in dolphins under human care ranged between 47.2–106.4 μg/ml, with a mean concentration of 64.1 ± 16.3 μg/ml. Serum concentration of Vanin-1 in free-ranging dolphins ranged between 31.0–70.5 μg/ml, with a mean concentration of 46.9 ± 11.7 μg/ml. Mean Vanin-1 concentration was significantly lower in free-ranging dolphins versus dolphins under human care (Fig. 2, T-test, P < 0.05). There was no significant difference between Vanin-1 levels in males versus females for either human-care or free-ranging dolphins (P > 0.3).

Correlations between routine clinical laboratory values and serum Vanin-1 concentration were determined for human-care and free-ranging populations separately. Clinical laboratory values (Supplemental Table 2) are consistent with the previously published cohort^26^ where human-care dolphins had lower WBCs, and higher triglycerides, cholesterol, GGT, and uric acid compared to the free-ranging dolphins. Unlike the previously published cohort, in this sub-cohort there was no difference in ALT or total iron which likely is due to the smaller sample size. Serum Vanin-1 levels were not correlated with any laboratory value in the human-care dolphin group; however, Vanin-1 was positively correlated with liver enzymes AST (ρ = 0.64) and ALT (ρ = 0.68) and inversely correlated with WBC (ρ = −0.68) and fibrinogen (ρ = −0.70) in the free-ranging dolphin group (Table 2). There was no correlation with age (Table 2, p > 0.2).

## Discussion

A considerable number of proteomic studies have focused on characterizing and comparing proteomes of body fluids from humans or model laboratory animals^25^,^27^,^28^,^29^,^30^, as well as determining depletion strategies to investigate lower abundance proteins^31^. Because protein identification by mass spectrometry is tied closely to species-specific genome availability, fewer studies have interrogated the differences in the body fluid proteomes between species^32^,^33^,^34^ as compared to examining proteomic differences due to experimental conditions within species. Further, peptide level variation within individuals is often not captured within reference genomes and intraspecific protein comparisons can be influenced by the diversity of the proteomic database used^35^. In the case of marine mammals, genomic, transcriptomic, and proteomic studies are all hindered by this limitation thereby resulting in incomplete comparative analyses that can reveal unique features underlying a unique physiology. For dolphins, incomplete or non-existent sequence data is an impediment to protein identification. In this study, immunoglobulins are under-annotated or predicted in the dolphin genome and, although they comprise a major component of the serum proteome, were unable to be included in the comparison between human and pig proteomes. In spite of incomplete biological databases, the information available can be useful for identifying extraordinary features of the proteome between species as demonstrated herein.

To advance the study of serum proteins in marine mammals, we surveyed the bottlenose dolphin serum proteome and utilized ranks to investigate potential outliers in a comparison with human undepleted proteomes. This analysis revealed 11 proteins that were identified only in the dolphin (Table 1). Although the dolphin serum proteins were not discovered in the pooled normal human sample, all of these 11 proteins have been observed in the human plasma proteome^25^.

Enrichment analysis^36^ of human orthologs relative to the 11 dolphin proteins resulted in only high-level gene ontology biological process classification and no significant network membership which suggests no known biological interaction amongst the serum proteins. Of the shared common features, all 11 proteins were classified at a lower level as members of; extracellular exosomes, which is reasonable given that most serum proteins are secreted into the blood. Human protein atlas classification of cell-type expression indicated that 9 of 11 proteins were expressed in adipocytes. Given that dolphins have a blubber layer; it is interesting to speculate that the serum protein differences relative to humans and pigs may have been influenced by this anatomical characteristic. Because Vanin-1 and Extracellular Matrix Protein 1 were not included in the human adipocyte classification, it would be premature to draw any conclusions without evidence for cell-specific expression in dolphins.

To account for differences due to phylogeny, comparisons were also made to an undepleted pig serum proteome, which is a terrestrial species that shared a more recent common ancestor with the dolphin. Of the 11 dolphin proteins identified as outliers (Fig. 1), three of these proteins; Fetuin B, Extracellular matrix protein 1, and Glycosylphosphatidylinositol phospholipase D1, were identified in undepleted pig serum and could represent proteins that are generally more abundant in taxa belonging to the order Cetartiodactyla. The inclusion of two fibrinogen chain proteins in the differentially ranked serum protein list (Table 1) is dubious and likely reflects differences in the sample preparation of serum because fibrinogen reference ranges in bottlenose dolphins are within reference ranges for other mammals^37^. Of the remaining six dolphin proteins that were not shared by human or pig proteome datasets, five of these proteins had high absolute rank differences (greater than 200) compared to the human plasma proteome and appear to be exclusive to the high abundance serum proteome of dolphins: Adiponectin, Vanin-1, Deleted in malignant brain tumors 1, Polymeric immunoglobulin receptor, and Adipocyte plasma membrane protein. Although Glutathione peroxidase 3 was not identified in the human or pig proteome datasets, compared to the more comprehensive human plasma proteome list, the rank difference in glutathione peroxidase 3 was not included amongst the high absolute rank difference group.

Protein rankings based on normalized spectral abundance factor provide a good estimate of relative protein abundance^38^, but are still susceptible to interferences such as matrix effects that can affect protein estimates. Targeted reaction monitoring assays^39^, such as parallel reaction monitoring, that include isotopically labelled peptide standards can greatly assist in reducing variability due to instrumentation or false-discovery artifacts of peptide assignment^40^ and provide a better absolute estimate of protein concentration. Parallel reaction monitoring was previously utilized to measure one of the 11 proteins, adiponectin, from Table 1. Dolphin (human-care and free-ranging) serum concentrations of adiponectin measured by reaction monitoring^22^,^26^,^41^ range between 2.3–47.9 μg/ml. Adiponectin concentrations, measured by reaction monitoring in humans, range between 0.02 μg/ml and 3 μg/ml^42^,^43^. These absolute differences between dolphin and human adiponectin are consistent with the rank relationship between dolphins and humans (Table 1).

To further verify differences between the lower abundance human homologs and higher abundance dolphin homologs, serum Vanin-1 was measured using parallel reaction monitoring in dolphins from two different populations. Serum from free-ranging dolphins and human-care dolphins was obtained from a previously published study^26^ that was conducted to characterize differences in metabolic phenotype between both populations. Serum Vanin-1 concentration in all dolphins was consistently high regardless of the population from which the animal belonged (Fig. 2) suggesting that a high Vanin-1 phenotype is a common feature of dolphins and not an artifact of management. Similar to adiponectin, Vanin-1 concentration in dolphins was also consistent with the relative ranking between dolphins and humans (Table 1). Serum concentrations in a cohort of renal transplant patients suggests Vanin-1 concentration is at least very low in both pre-operative (1.9 ng/ml) and post-operative (3.7 ng/ml) patients measured by ELISA^44^. Substantially higher concentrations (~20–200 ng/mg protein or ~1.7–17.7 μg/ml) were measured using an ELISA from the same manufacturer in a study of normal volunteers and patients with primary immune thrombocytopenia. Despite these highly variable estimates in normal humans, Vanin-1 levels in dolphins appear to be 20–1000 times higher than human. Because reported human serum measurements from the same ELISA manufacturer were incongruent^44^,^45^ with estimates based on proteomic analysis^25^, we performed a product ion scan targeting the homologous human Vanin-1 peptide utilized to measure dolphin Vanin-1 in this study. No signal for the human Vanin-1 peptide was found in normal pooled human serum (data not shown) suggesting human Vanin-1 levels are indeed low and likely to be in the range of ng/ml.

Vanin-1 is a membrane-bound GPI-anchored^46^,^47^ pantetheinase expressed widely, but primarily in liver, small intestine, and kidney in humans^48^. As with other GPI-anchored proteins, it is predicted that Vanin-1 can be cleaved by glycosylphosphoinositol phospholipases and released into the circulation. Interestingly, Glycosylphosphoinositol phospholipase D1 was one of the 11 proteins in Table 1 that were elevated in rank compared to human and this protein is known to cleave GPI-anchored proteins^49^. Because Glycosylphosphoinositol phospholipase D1 was also identified from pig serum, the elevated level of Vanin-1 in dolphin serum may have resulted due to elevated expression of Vanin-1, elevated activity of Glycosylphosphoinositol phospholipase D1, or possibly a combination of expression and activity. The gene product of *Vanin-1* catalyzes the conversion of pantetheine to cysteamine and pantothenate (vitamin B5). Whereas pantothenate is recycled back to acetyl-coenzyme A, the free thiol generated in this reaction, cysteamine, has attracted much attention due to its potential oxidative and antioxidative properties^50^. The history of research involving Vanin-1 and cysteamine presents a very complicated biological picture with many opposing conclusions. Addition of cysteamine at low concentration to leukocytes promotes H_2_O_2_-induced DNA damage; whereas, at high concentration cysteamine prevented DNA damage^51^. Early studies of cysteamine were primarily focused on this product as a thiol-mediated mechanism of H_2_O_2_ generation which promoted cell toxicity^52^. Fueling the idea of cysteamine as an injurious thiol, Vanin-1 knock-out mice lacking measureable tissue cysteamine exhibited a protective phenotype when challenged with gamma-irradiation or paraquat^53^. Furthermore, popular rodent models of duodenal ulcers are created through the administration of cysteamine^54^,^55^. On the other hand, Vanin-1 confers a protective phenotype to pancreatic beta islet cells^56^, hepatotoxic liver injury^57^, and red blood cells^58^ while cysteamine administration has been shown to reduce renal fibrosis^59^, renal cystinosis^60^, and neurodegenerative disorders^61^.

Although speculative, the elevation in dolphin serum Vanin-1 may lead to an enhancement of Selenium-independent glutathione peroxidase activity. In Vanin-1 knock-out mice, Selenium independent glutathione peroxidase activity in liver, thymus and testes is reduced to about one half the level of wild type mice, and administration of cysteamine restored activity to wild-type levels^62^. Selenium-dependent glutathione peroxidase activities are elevated in marine mammal tissues and plasma and have been implicated as a protective mechanism to counter ischemia/reperfusion injury due to diving^6^,^12^. Interestingly, glutathione peroxidase 3 was one of the top differentially ranked proteins in Table 1, but because it is a selenium-dependent glutathione peroxidase, the link between Vanin-1 and cysteamine is not supported. Because studies of selenium-independent glutathione peroxidase activity in marine mammals is lacking, the association between glutathione peroxidase activity in marine mammals and elevated Vanin-1 in the serum remains a potentially interesting question that may explain part of the high antioxidant status in marine mammal plasma^6^. Alternatively, because cysteamine at high concentrations has been reported to be protective only in cells under high oxidative stress^63^, it is interesting to speculate that high Vanin-1 levels may have evolved in response to counter the oxidative stress due to ischemia/reperfusion due to diving.

In addition to the finding that all study dolphins have high circulating levels of Vanin-1, the finding that dolphins under human care had slightly elevated (1.3 fold) serum Vanin-1 concentration was unexpected (Fig. 1). Because serum Vanin-1 levels are prone to elevate as a result of fasting^64^,^65^, the higher Vanin-1 levels in the dolphins under human care seemed unlikely because all human care dolphin samples were drawn two hours after feeding and all dolphins had evidence of having recently fed due to the presence of stomach contents detected via ultrasound. Gut content was not determined for the free-ranging dolphins, but these dolphins are known to feed frequently throughout the day^66^. It remains possible that the dolphins under human care had elevated levels of Vanin-1 as a result of an overnight fast that preceded a scheduled feeding and blood draw and that Vanin-1was actively declining. Alternatively, the higher serum Vanin-1 levels in human-care dolphins may help to explain part of the reason why this population of dolphins has reportedly higher serum levels of glucose, insulin, triglycerides, and incidence of hepatic steatosis compared to the free-ranging population^26^,^67^. Overexpression of Vanin-1 leads to enhanced hepatic gluconeogenesis, elevated blood glucose, and insulin resistance in C57BL/6 mice^64^ Further, the knock-down of Vanin-1 in db/db mice, which have elevated *Vanin-1* expression and are prone to hepatic steatosis, led to the attenuation of hepatic steatosis^64^. The inhibition or knock-out of Vanin-1 leads to an increase in hepatic triglyceride level in fasted rats or mice^65^ and improves glucose tolerance and insulin sensitivity in mice fed a high fat diet^68^. Recent studies, however, suggest that acute inhibition of Vanin-1 activity in Zucker Diabetic Fatty rats did not change the degree of steatosis nor did it affect insulin sensitivity or glucose production^68^. AlthoughVanin-1 plays a role in hepatic fatty acid oxidation, and that deviation to either side of normal may lead to steatosis, the contribution of Vanin-1 to the promotion of steatosis and insulin sensitivity in non-laboratory models is less clear.

To determine whether Vanin-1 correlates with clinical laboratory values of hepatic function, we performed a Pearson product moment correlation for both populations (Table 2). Due to the small sample size, the correlation analysis was underpowered and any associations not considered significant should be tested independently using a larger population. To detect a significant (α = 0.05) correlation of 0.4 at a power of 0.80, approximately 46 dolphins would have needed to have been included in the analysis. Therefore, only strong correlations were able to be detected and lack of correlation with clinical variables in this study should be interpreted with caution. In the free-ranging population, Vanin-1 levels were positively correlated with the liver enzymes ALT and AST, and negatively correlated with fibrinogen which is synthesized primarily in the liver. The moderate correlation with circulating liver enzyme levels suggest that serum Vanin-1 may be elevated in free-ranging dolphins with liver dysfunction; a common pathological finding in stranded dolphins^69^. However, no correlation between Vanin-1 and any clinical blood chemistry value was made for the human-care population from which hepatic steatosis^67^ is also known to be prevalent. Generalizations regarding serum Vanin-1 levels and clinical correlates of health require a much larger time-controlled study to determine whether differences in serum Vanin-1 can be of value in predicting liver health status in dolphins.

In conclusion, a simple proteomic analysis of bottlenose dolphin serum revealed a suite of circulating proteins that appear to be elevated compared to normal humans and a closer terrestrial ancestor, pig. Absolute protein measurements supported the ranking data and led to the discovery that serum Vanin-1 levels are consistently and comparatively high in dolphins. Basic proteomic comparison between other marine mammals and terrestrial mammals should help to unveil significant alternative biochemical adaptations to stress protection and metabolism in marine mammals which should be incorporated in future studies.

## Methods

### Serum samples

The collection of blood from bottlenose dolphins under human care was performed in accordance with guidelines and regulations set forth by the Navy Marine Mammal Program Institutional Animal Care and Use Committee (IACUC) and the Navy Bureau of Medicine (Protocol #101–2012). The collection of blood from free-ranging dolphins was performed in accordance with guidelines and regulations set forth by the Mote Marine Laboratory IACUC under National Marine Fisheries Service Scientific Research Permit No. 522–1785. The collection of pig blood was performed in accordance with guidelines set forth by the Medical University of South Carolina IACUC under protocol number 3234.

For proteomic comparisons, sera from six bottlenose dolphins (*Tursiops truncatus*) under human care were previously collected and information regarding these samples was previously described^70^. Briefly, serum samples were collected at 6 time points (0, 3, 6, 12, 18, 24 weeks) during a feeding study. Baseline samples were drawn at day zero and subsequent samples were drawn after the diet was modified to include pinfish and/or mullet. Dolphins included three females aged 12, 32, and 52 years, and three males aged 8, 35, and 42 years. Blood was drawn two hours after feeding from the peduncle using a 19 or 21 gauge needle and collected in a BD Vacutainer serum separator tube. Whole blood was allowed to clot for 30–60 minutes and centrifuged at 1,100 × g for 10 minutes prior to being transferred to a cryovial for storage at −80 °C.

Yorkshire pig (*Sus scrofa*) serum was collected from clotted whole blood during routine surgery. Blood was drawn from an ear vein and transferred to a 1.5 ml Eppendorf ^TM^ protein Lobind tube. Blood was allowed to clot for 30 minutes at room temperature and centrifuged for 10 minutes at 1,500 × g at 4 °C. Serum was removed from the clot and stored at −80 °C.

Pooled normal human serum was purchased from Innovative Research (Novi, MI; IPLA-SER). According to the distributor’s protocol, 500–600 mLs whole blood is collected in dry bleed bags without anticoagulant from human volunteers. Whole blood is centrifuged immediately between 1 °C and 4 °C. Plasma is extracted from red blood cells and allowed to clot for 48 hours at room temperature. Clotted plasma is centrifuged for 10 mins at 5000 × g for 20 minutes. Serum is removed off the clot and stored at −80 °C.

For Vanin-1 measurements by parallel reaction monitoring. a subset of frozen serum samples were analyzed from “managed” dolphins under human-care (N = 10) and free-ranging dolphins sampled during capture-release health assessments^71^ (N = 10). These samples were selected from a repository of samples described in a previously published study^26^. Dolphins were matched for age and sex and blood draws were within one year of each other. Dolphin serum samples under human-care were collected exactly as described for dolphin serum samples utilized for proteomic studies. Whole blood from free-ranging dolphins was drawn from a highly visible vessel running through the middle of the ventral fluke using 19 gauge, ¾ inch needles connected to BD vacutainer serum separator tubes. Whole blood was centrifuged at 1,800 × g for 10 minutes at room temperature. Serum was removed from the clot, placed into a cryovial, and stored at −80 °C.

Herein, dolphins that were under human care will be referred to as such or as human-care dolphins.

### Clinical laboratory data

All clinical laboratory methods for dolphins under human care and free-ranging dolphins were previously described^26^.

### Proteomics

Proteomic analysis of the six dolphin serum samples at six different time points was previously reported in Sobolesky *et al*.^22^. Data deposited in the ProteomeXchange Consortium (http://proteomecentral.proteomexchange.org) via the PRIDE partner repository^72^ with the dataset identifier (PXD003425) were utilized for creating protein rankings. Proteomic analyses of pig and human sera followed a similar procedure described herein. Five microliters serum from pig or human was aliquoted and digested with trypsin accordingly^22^. Peptides were extracted with solid-phase cartridges (Phenomenex, Strata-X 33 μ, 60 mg) and eluted with 40% acetonitrile in 0.1% formic acid. Samples were dried by speed-vac and resuspended in mobile phase A [98% water/2% acetonitrile/0.1% formic acid]. Peptides were trapped onto a 100 μm × 1 cm C18 (100 Å with 5-μm particles) trap column (Acclaim PepMap 100; Thermo Fisher Scientific) and separated with a 75 μm × 15 cm C18 (100 Å with 3-μm particles) analytical column (Acclaim PepMap 100; ThermoFisher Scientific). Flow rate was set to 350 nl/min and peptides were eluted using a gradient from 5% to 40% mobile phase B [5% water/95% acetonitrile/0.1% formic acid] over 60 minutes on a 2D + NanoLC system (Eksigent, Dublin, CA). Mass spectrometry data were acquired with a TripleTOF 5600 System (SCIEX, Foster City, CA) with nanospray source. Source parameters were set to the following: temperature = 150 °C, source voltage = 2500 V, GS1 = 2; Curtain gas = 15; declustering potential = 100 V; collision energy = 10 V. Data were acquired in positive ion mode using information dependent acquisition. The TOF mass range was set between 350–1250 m/z. Precursor ion scans were acquired for 200 ms. Product ion scans were acquired for 50 ms for + 2 to + 5 charged parent ions. Up to 20 product ion scans were selected per cycle and excluded for 5 seconds if previously selected. Raw peptide data (.wiff) were converted to Mascot generic file format (.mgf) using the SCIEX MS Data Converter (v. 1.3 beta, July 2012). Protein identifications were made using Mascot (v. 2.4.1, Matrix Science). Dolphin data were searched against the Ensembl (release 64) turTru1 dolphin genome assembly protein database (16,598 sequences^73^). Pig data were searched against the reviewed (Swiss-prot) and unreviewed (Trembl) databases (release 2015_12, 26,148 protein sequences). Human data were searched against the Swiss-prot database (Release 2015_12, 20,199 protein sequences) and Swiss-prot isoform database (Release 2015_12, 21,920 protein sequences). All database searches included the common Repository of Adventitious Proteins database (cRAP; 2012.01.01; the Global Proteome Machine). The following MASCOT parameters were selected: enzyme = trypsin; max missed cleavages = 2; carbamidomethylation (Cys) was specified as a fixed modification; oxidation (Met) and pyro-glu (N-term Q) were selected as variable modifications; precursor ion tolerance = 20 ppm; fragment ion tolerance = 0.1 Da; instrument type was set to ESI-QUAD-TOF. Mascot search results were uploaded to Scaffold 4Q + (Proteome Sciences) and normalized abundance factor calculated. False discovery rate was limited in Scaffold 4Q + to 1% for both peptides and proteins. Human and pig protein identifications required two peptides. Dolphin proteins required three identified peptides to ensure a high level of confidence in the protein identification thereby making any downstream rank comparison more conservative. Proteins that did not have an identifying name in the dolphin or pig FASTA database were manually annotated by similarity comparison using BLAST and manually annotated names reflect those relevant to human proteins.

### Protein rank comparisons

Protein lists were exported to a spreadsheet and contaminant proteins and immunoglobulins were removed from all species due to the lack of immunoglobulin sequence data in the dolphin proteome FASTA database. Proteins were ranked based on normalized spectral abundance factor (NSAF)^74^ and ranks were compiled between orthologous dolphin and human proteins. Mean ranks were calculated for the six dolphin species at each time point (0, 3, 6, 12, 18, and 24 weeks) and reordered from highest to lowest. Arithmetic mean ranks were chosen over median ranking or geometric mean ranking in accordance with the previously reported rationale for ranking of high abundance serum proteins for humans^23^. The absolute value of the difference between the dolphin and human serum protein rank was calculated and plotted (Fig. 1). For dolphin proteins that were not discovered in the human serum proteome analysis, individual comparisons were made against the NSAF ranked pig serum proteome that was ranked in an identical manner as the dolphin and human protein datasets. Further, dolphin proteins were also compared against the ranked human plasma proteome^25^. The previously published dataset from the human plasma proteome was sorted highest to lowest based on estimated plasma concentration after immunoglobulins and keratins were removed. Complete protein lists and rankings are provided in Supplemental Table 1.

### Parallel reaction monitoring

For parallel reaction monitoring, a synthetic peptide was constructed (New England Peptide, Gardner, MA) pertaining to amino acids 159–172 of dolphin Vanin-1 (ENSTTRP00000000565) with incorporation of ^13^C/^15^N lysine at the c-terminus: YQYNTDVVFDSEGK^. The peptide was selected because it did not contain methionine or cysteine, did not begin with a glutamine residue, and was not in a domain that is known to be modified in mammals according to Uniprot annotation. As such, only a single tryptic peptide met all criteria for dolphin Vanin-1. Serum samples were prepared as described for proteomic analysis and followed procedures described in ref. 22. Five μls serum was digested with trypsin (Trypsin gold, Promega) at a 1:10 ratio in 50 mM ammonium bicarbonate/0.1% anionic acid-labile surfactant (Protea Bioscences, Morgantown. WV). Prior to solid phase extraction, 6 pmols peptide standard was spiked into the digest resulting in a total of 150 fmols injected on column. All chromatography and acquisition parameters were the same as described above with the exception that product ion collision energy was set to 36. The y12^+1^ ion was extracted for quantification of the native peptide (832.9 −>1373.62 ± 0.1 m/z) and standard peptide (836.9 −>1381.62 ± 0.1 m/z). An external calibration curve from 0.78 fmol/μl to 150 fmol/μl was constructed by spiking the isotope labelled standard peptide in dolphin serum protein trypsin digest matrix. Quantitative measurements were calculated by area under the curve for ion pairs in MultiQuant (v 2.0.2, AB Sciex). An experimental blank was processed in triplicate to calculate the limit of detection (LOD) and limit of quantification (LOQ) for the stable isotope labeled peptide on the day of the analytical run. Regression was performed using a linear fit with 1/x weighting (y = 1729.95x + 942.35, *r *= 0.997). LOD was calculated as 3σ/slope of the blank and LOQ was calculated as 10σ/slope^75^. Assay variability was determined by injecting two samples in triplicate at the beginning, middle and end of the run and %RSD was calculated.

### Statistics

Clinical and analytical data were tested by T-test if data had normal distributions or Mann-Whitney U test for non-normally distributed data. Normality was tested using the Shapiro-Wilk test and equal variance tested using the Equal Variance Test in Sigmaplot version 11.0. Significance was defined as P < 0.05. Correlations were determined using Pearson’s product moment. All data are expressed as mean ± standard deviation unless otherwise noted.

### Enrichment analysis

Orthologous human protein identifiers were used in place of dolphin protein identifiers for enrichment analysis. Proteins were classified by Gene ontology (GO) using WebGestalt^36^ with the following criteria: Hypergeometric statistical method; Benjamini-Hochberg multiple testing; Significance level 0.05; Minimum Number of Genes per Category = 5. Cell-specific expression was categorized using the Human Protein Atlas^76^ database through the gene profiling tool G-Profiler^77^ using the following criteria: Significant only selected; Size of query = 3; Significance threshold = Benjamini-Hochberg FDR.

### Data availability

Human and Pig proteomic data is available via ProteomeXchange with identifier PXD004967.

## Additional Information

**How to cite this article**: Sobolesky, P. *et al*. Proteomic Analysis of Non-depleted Serum Proteins from Bottlenose Dolphins Uncovers a High Vanin-1 Phenotype. *Sci. Rep.* **6**, 33879; doi: 10.1038/srep33879 (2016).

## Supplementary Material

Supplementary Table S1

Supplementary Table S2

Supplementary Table S3

Supplementary Table S4

## Figures and Tables

**Table 1:** Mean ranks of eleven dolphin serum proteins not present in the undepleted pooled human serum proteome results.

Protein Name	ENSEMBl Accession Number	MW	Identified in all 6 dolphins at any time	Dolphin serum	Human serum	Pig serum	Human (Farrah *et al*.^25^)plasma	
				Mean Rank (±SD)	Rank	Rank	Rank	Est. conc.(ng/ml)
Fetuin B	ENSTTRP00000015959	41 kDa	Yes	13 ± 1	—	57	185	270
Glutathione peroxidase 3	ENSTTRP00000005431	26 kDa	Yes	27 ± 4	—	—	69	10000
Adiponectin	ENSTTRP00000015964	26 kDa	Yes	30 ± 9	—	—	265	120
Vanin 1	ENSTTRP00000000565	57 kDa	Yes	36 ± 4	—	—	348	56
Fibrinogen beta chain	ENSTTRP00000009538	56 kDa	No	44 ± 19	—	—	18	130000
Fibrinogen gamma chain	ENSTTRP00000011681	90 kDa	No	47 ± 17	—	—	21	98000
Extracellular matrix protein 1	ENSTTRP00000010082	62 kDa	Yes	47 ± 3	—	73	131	770
Deleted in malignant brain tumors 1	ENSTTRP00000004960	173 kDa	Yes	47 ± 4	—	—	1176	N/A
Polymeric immunoglobulin receptor	ENSTTRP00000011489	83 kDa	Yes	50 ± 4	—	—	465	25
Adipocyte plasma membrane protein	ENSTTRP00000001536	46 kDa	No	51 ± 6	—	—	379	29
Glycosylphosphatidylinositol phospholipase D1	ENSTTRP00000000260	90 kDa	No	56 ± 3	—	76	155	460

Protein ranks for orthologous proteins from the undepleted pig serum proteome and the well characterized human plasma proteome, along with estimated concentrations (Est. Conc.) are provided. MW = predicted molecular weight in kilodaltons. A horizontal dash indicates that no protein was identified. A ‘No’ in the fourth column indicates that proteins were not consistently identified for every dolphin at each time point in the 24 week study^21^.

**Table 2:** Pearson’s product moment correlation between blood clinical data and serum Vanin-1.

		Wild	Managed
ALT	Pearson’s r	0.68	0.24
	p-value	0.03	0.51
AST	Pearson’s r	0.64	−0.12
	p-value	0.05	0.74
WBC	Pearson’s r	−0.68	0.17
	p-value	0.03	0.63
Fibrinogen	Pearson’s r	−0.70	ND
	p-value	0.02	
Age	Pearson’s r	−0.39	−0.40
	p-value	0.26	0.25

Significant associations were accepted if P < 0.05. No associations were found between serum Vanin-1 and laboratory blood values.
